# Supplementary material for: Deletions in CWH43 cause idiopathic normal pressure hydrocephalus
Source: EMBO Mol Med. 2021 Jan 18;13(3):e13249. doi: 10.15252/emmm.202013249 (PMC7933959; doi:10.15252/emmm.202013249)
Supplement: Supplementary file 1 — Appendix [file EMMM-13-e13249-s001.pdf]

Appendix

Deletions in *CWH43* Cause Idiopathic Normal Pressure Hydrocephalus

Hong Wei Yang[1,3,4], Semin Lee[3,4], Dejun Yang[1], Huijun Dai[1], Yan Zhang[1], Lei Han[3,4], Sijun Zhao[1], Shuo Zhang[1], Yan Ma[1], Marciana F. Johnson[3,4], Anna K. Rattray[3,4], Tatyana A. Johnson[3,4], George Wang[1,3,4], Shaokuan Zheng[1], Rona S. Carroll[1,3,4], Peter J. Park[3,4] and Mark D. Johnson[1,2,3,4, *]

[1]University of Massachusetts Medical School, Worcester, MA 01655
[2]UMass Memorial Health Care, Worcester, MA 01655
[3]Brigham and Women's Hospital, Boston, MA 02115
[4]Harvard Medical School, Boston, MA 02115

*Correspondence:
Mark D. Johnson, MD, PhD
Department of Neurological Surgery
University of Massachusetts Medical School
UMass Memorial Health Care
55 Lake Avenue North
Worcester, MA 01655
Phone: 508-334-0605
FAX: 508-856-5074
Email: mark.johnson3@umassmemorial.org

Content

Appendix Figure S1. Screen shot of sequencing data for *CWH43*: NM_001286791:exon16:c.2005delA: p.K696fs.

Appendix Figure S2. Screen shot of sequencing data for *CWH43*:NM_001286791:exon12: c.1515delA: p.Leu533Ter.

Appendix Figure S3. iNPH patients harboring *CWH43* deletions have larger cerebral ventricles than asymptomatic age and gender-matched controls.

Appendix Figure S4. iNPH-associated *CWH43* mutation decreases vesicular association but not cell surface expression of CD59.

Appendix Figure S5. Cwh43 protein is highly expressed in mouse choroid plexus.

Appendix Figure S6. CRISPR/Cas9 generation of mutant *CWH43* alleles in mice.

Appendix Figure S7. *CWH43* mutant mice develop communicating hydrocephalus.

Appendix Figure S8. Loss of Cwh43 immunoreactivity in the choroid plexus of *CWH43*$^{M533/M533}$ mice**.**

Appendix Figure S9. *CWH43* mutation decreases cilia number in *CWH43*$^{M533/A530}$ mice.

CWH43:NM_001286791:exon16:c.2005delA:p.K696fs

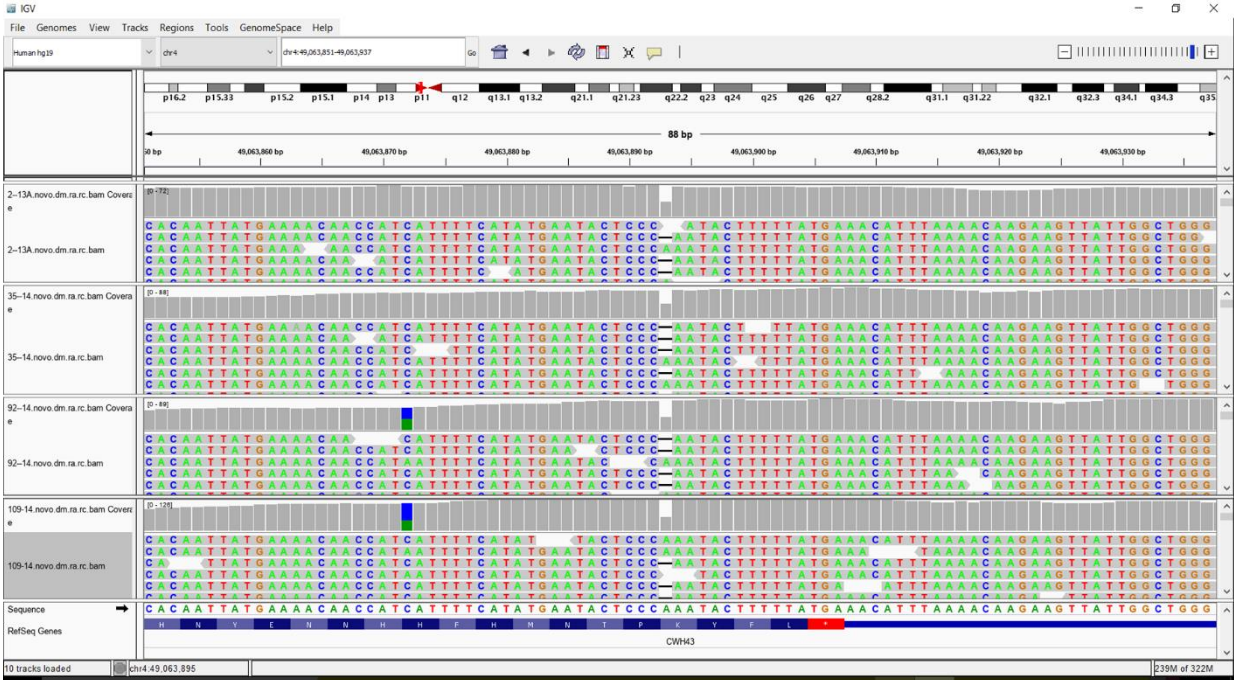

Appendix Figure S1. Screen shot of sequencing data for *CWH43*: NM_001286791:exon16:c.2005delA: p.K696fs.

Interactive Genome Viewer visualization showing reads used to call this variant. Data shown are re-assembled reads produced by GATK HaplotypeCaller–bamOutput. The data accurately represent what HaplotypeCaller was seeing when it called this variant.

CWH43:NM_001286791:exon 12:c.1515delA:p.Leu533Ter

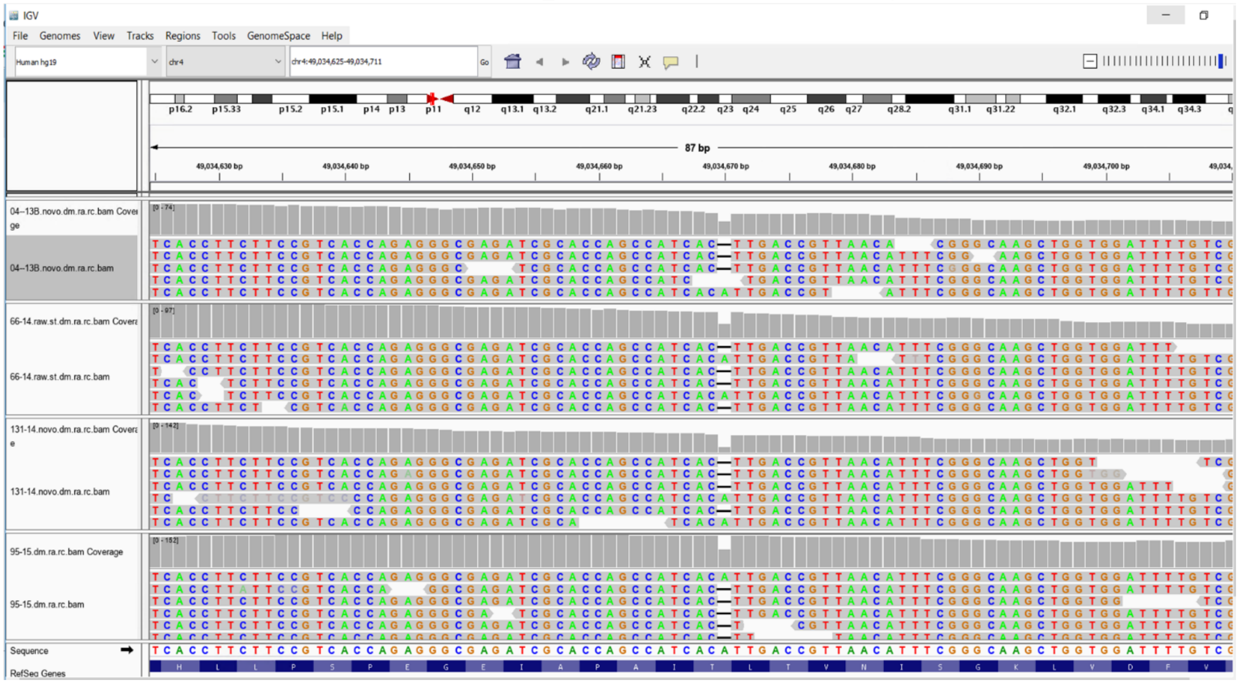

Appendix Figure S2. Screen shot of sequencing data for *CWH43*:NM_001286791:exon 12: c.1515delA: p.Leu533Ter.

Interactive Genome Viewer visualization showing reads used to call this variant. Data shown are re-assembled reads produced by GATK HaplotypeCaller –bamOutput. The data accurately represent what HaplotypeCaller was seeing when it called this variant.

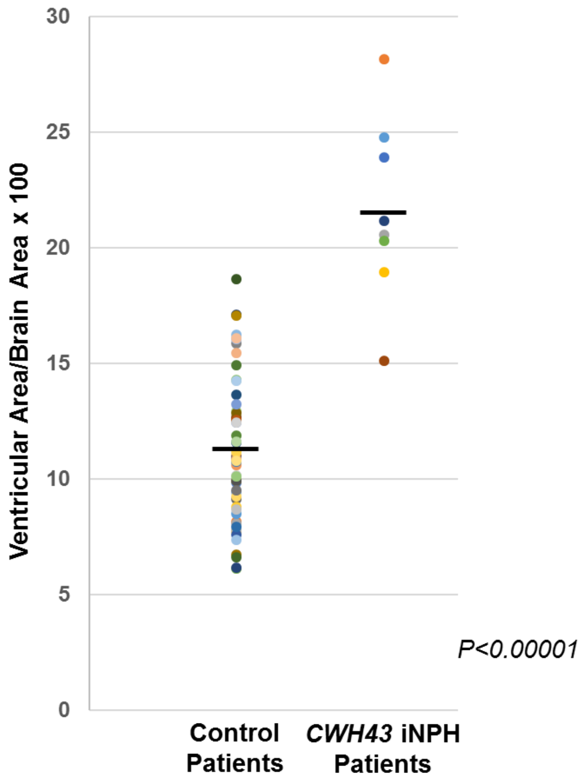

Appendix Figure S3. iNPH patients harboring *CWH43* deletions have larger cerebral ventricles than asymptomatic age and gender-matched controls.

Axial T2-weighted MR images of the brain were obtained from 8 iNPH patients harboring *CWH43* deletions and 54 asymptomatic individuals matched for age and gender (www.oasis-brains.org). Ventricular cross sectional area and brain cross sectional area from the same axial image were measured for each case, and the ratio of these measurements was obtained. Comparison of the two groups revealed that the mean ratio representing the normalized ventricular size among the 8 iNPH patients with *CWH43* deletions (21.7 ± 1.4) was significantly larger than that of the age and gender matched cohort of asymptomatic individuals (11.3 ± 0.4; $P<0.00001$, two tailed t-test). Horizontal bars represent the mean.

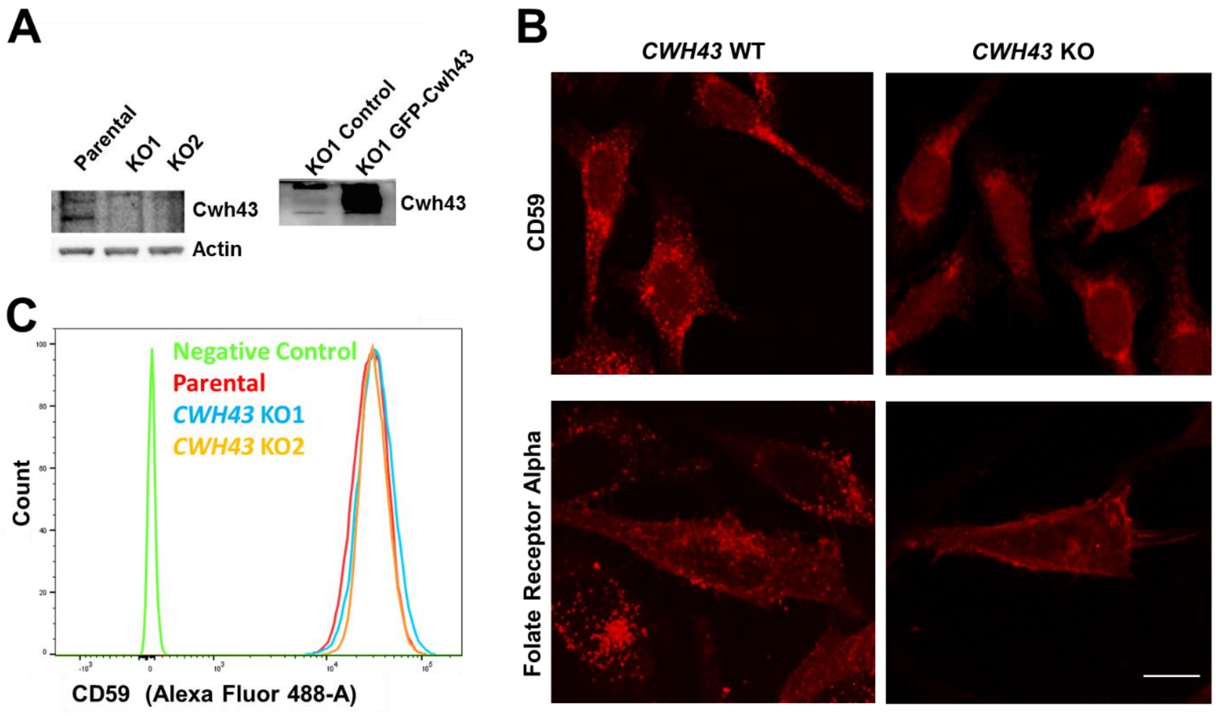

Appendix Figure S4. iNPH-associated *CWH43* mutation decreases vesicular association but not cell surface expression of CD59.

A. *left panel* - Western blot showing loss of Cwh43 protein expression in two independent HeLa cell lines (KO1 and KO2) in which the *CWH43* gene has been disrupted at Leu533 using CRISPR/Cas9 technology. *right panel*
-Transient overexpression of GFP-Cwh43 in *CWH43* KO HeLa cells, followed by Western blot for Cwh43.

B. Fluorescence micrographs showing subcellular localization of two GPI-anchored proteins in wild type (parental) or *CWH43* knockout (*CWH43* KO) HeLa cells. Cells were transiently transfected with plasmids encoding human RFP-CD59 or RFP-folate receptor alpha fusion proteins. Scale is approximately 5 μm.

C. Flow cytometry data showing cell surface CD59 expression in parental (wild type) and *CWH43* knockout HeLa cell lines. Cells were labeled using an anti-CD59 antibody conjugated to a fluorophore.

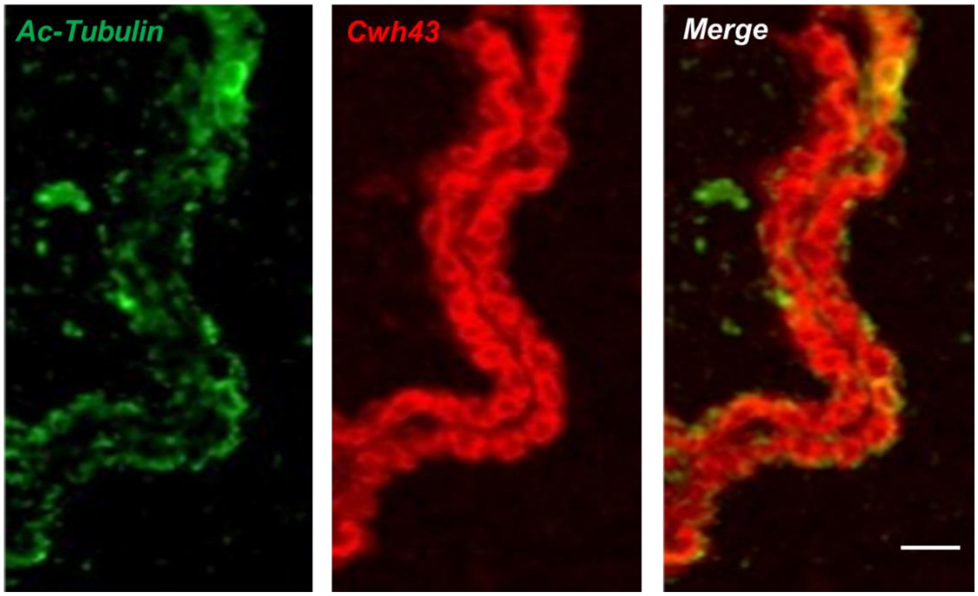

Appendix Figure S5. Cwh43 protein is highly expressed in mouse choroid plexus.

Immunofluorescence immunohistochemistry of the choroid plexus of the lateral ventricle of the mouse brain. Cilia are visualized using an antibody for acetylated alpha tubulin (green). Cwh43 is visualized using a specific anti-Cwh43 antibody (red). The yellow staining indicates the co-staining in the merged image. Scale is approximately 25 µm.

CWH43 Wild Type and CRISPR/Cas9 *CWH43* Mutant Alleles

WT

ATGGTGCTGTCTCGGTACCCGATTGTCAGATCGGAACATCACCTTCTTCCGTCGCCAGAGGGCGA
GATCGCACCAGCCATAACCATGACAGTTAACGTCTCCAACAGACTGGTGGATTTTGTGGTGACACA
CTTTGGGAATCATGA

Met V L S R Y P I V R S E H H L L P S P E G E I A P A I T **Met** T V N V S N R L V D F V V T H F G N H

CA/G; Met533Ter

ATGGTGCTGTCTCGGTACCCGATTGTCAGATCGGAACATCACCTTCTTCCGTCGCCAGAGGGCGAGA
TCGCACCAGCCATAACGTGACAGTTAACGTCTCCAACAGACTGGTGGATTTTGTGGTGACACACTTT
GGGAATCATGA

Met V L S R Y P I V R S E H H L L P S P E G E I A P A I T **Stop** Q L T S P T D W W I L W **Stop** H T L G I **Met**

ACCAGCCATA del; Ala530fs

ATGGTGCTGTCTCGGTACCCGATTGTCAGATCGGAACATCACCTTCTTCCGTCGCCAGAGGGCGA
GATCGCACCAGCCATAACCATGACAGTTAACGTCTCCAACAGACTGGTGGATTTTGTGGTGACACA
CTTTGGGAATCATG

Met V L S R Y P I V R S E H H L L P S P E G E I A P **Stop** Q L T S P T D W W I L W **Stop** H T L G I **Met**

Appendix Figure S6. CRISPR/Cas9 generation of mutant *CWH43* alleles in mice.

CRISPR/Cas9 and *CWH43* guide RNA injection into mouse embryos was used to generate two independent lines of C57bl6 *CWH43* mutant mice. Analysis of mouse DNA using RT-PCR and Sanger sequencing confirmed a 1 bp deletion in mouse *CWH43* (Met533Ter) corresponding to human 4:49034669 CA/C; Leu533Ter. A second mouse line (*CWH43*$^{M533/A530}$) harboring one *CWH43*^{M533} allele and a second *CWH43* allele with a 10 bp deletion that generates a stop codon and termination of Cwh43 at A530.

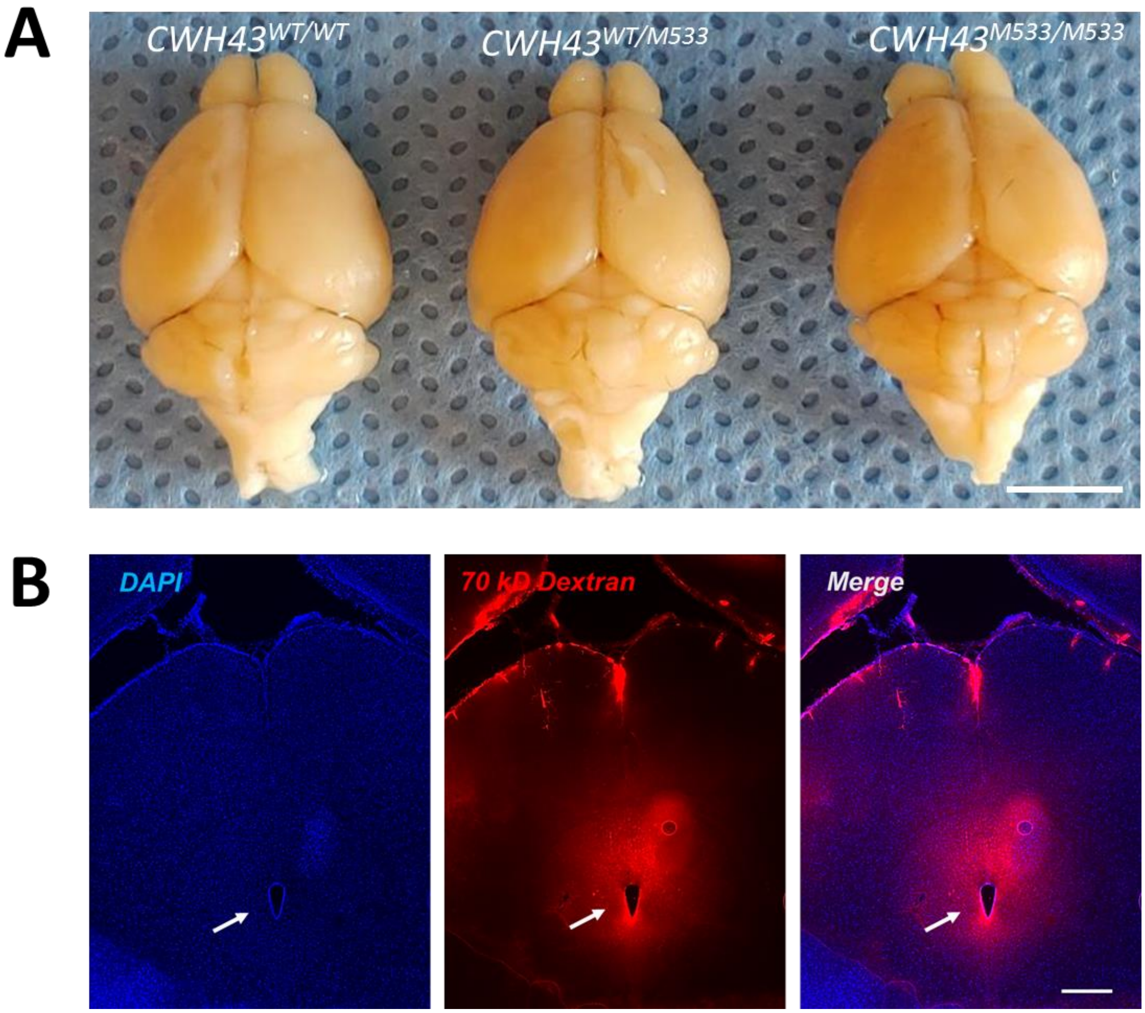

Appendix Figure S7. CWH43 mutant mice develop communicating hydrocephalus.

A. Images of the whole brain obtained from *CWH43* $^{WT/WT}$, *CWH43*$^{WT/M533}$, and *CWH43*$^{M533/M533}$ 12 months old mice. Scale is approximately 0.5 cm

B. Fluorescence micrographs of cryostat sections of a brain from a *CWH43*$^{M533/M533}$ mouse obtained at the level of cerebral aqueduct and periaqueductal gray (arrow). The brain sections have been stained with DAPI to identify cell nuclei (blue). Prior to harvesting the brain, the lateral ventricle was injected with 5 µl of saline containing a fluorescent dextran (70 KD). After 5 minutes, the brain was harvested and processed for fluorescence microscopy. Fluorescent dextran completely filled the ventricular system and paravascular spaces in the cortex, confirming patency of the cerebral aqueduct. Scale is approximately 5 mm.

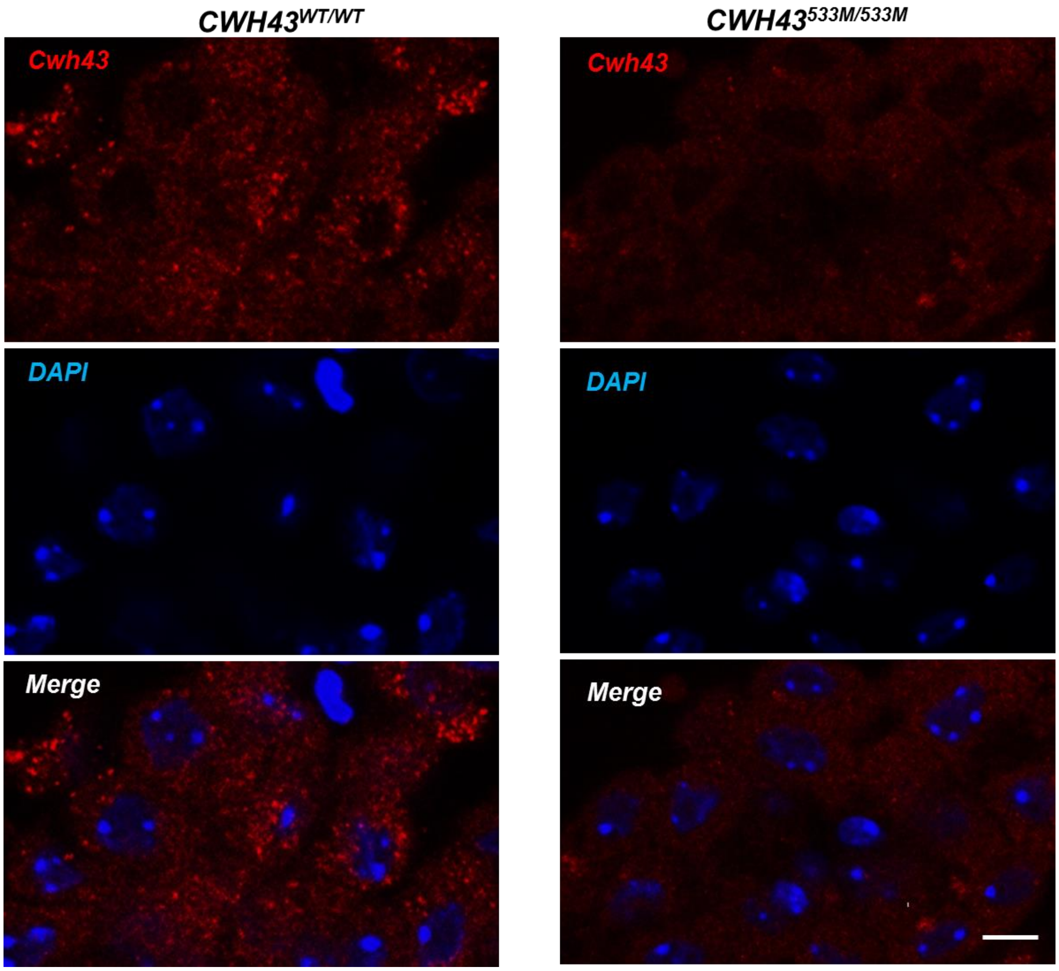

Appendix Figure S8. Loss of Cwh43 immunoreactivity in the choroid plexus of *CWH43*$^{M533/M533}$ mice.

Confocal immunofluorescence images showing Cwh43 immunoreactivity (red) in mouse choroid plexus obtained from *CWH43*$^{WT/WT}$ and *CWH43*$^{M533/M533}$ mice. Nuclei are counterstained with DAPI (blue). Scale is approximately 10 µm.

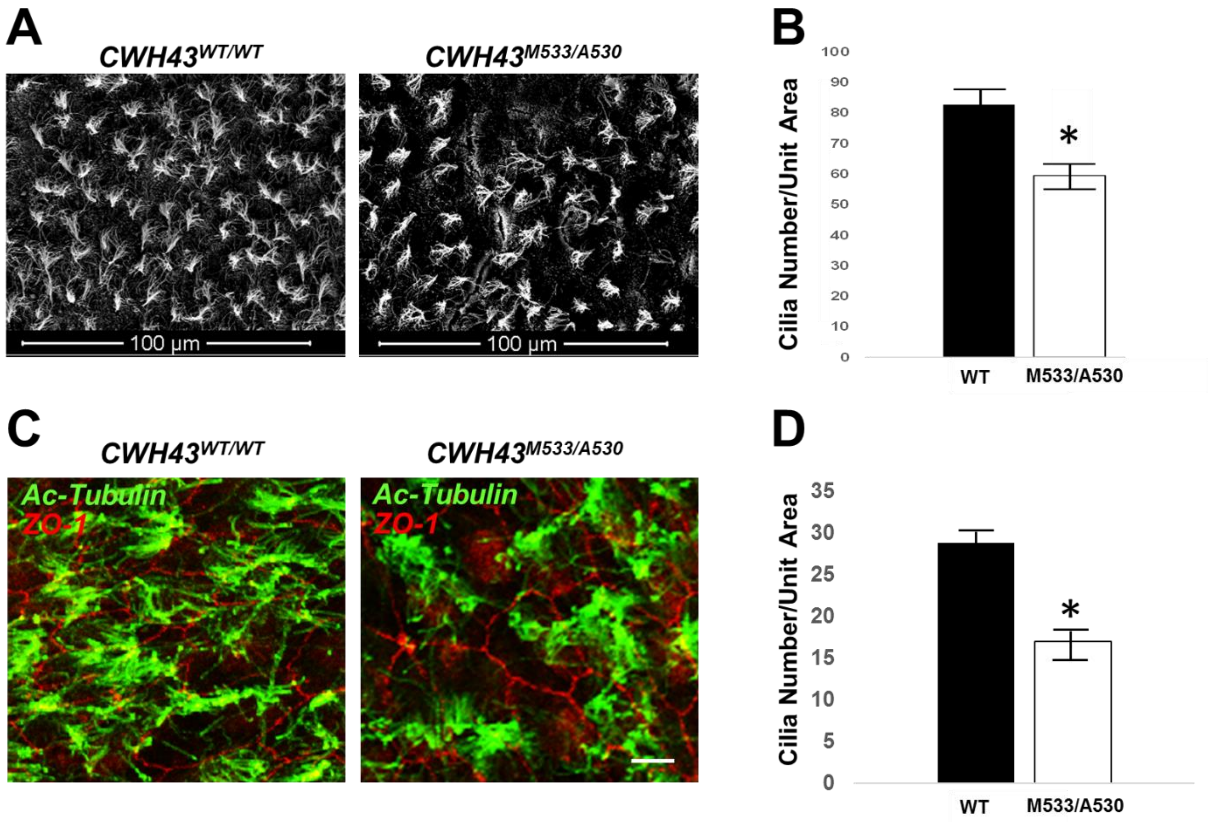

Appendix Figure S9. *CWH43* mutation decreases cilia number in *CWH43*$^{M533/A530}$ mice.

A. Scanning electron micrographs of the ependymal surface of the lateral ventricle of *CWH43*$^{WT/WT}$ and *CWH43*$^{M533/A530}$ mice. Scale is approximately 100 µm.

B. Quantitation of the cilia number/unit area from the electron micrographs on the left. Data shown are the mean ± SEM. * = P<0.0037, n = 4, unpaired t-test.

C. Immunofluorescence immunohistochemistry of the ependymal surface of the lateral ventricle of the mouse brain. Cilia are visualized using an antibody for acetylated alpha tubulin (green). The tight junction protein, ZO-1 is visualized using a specific anti-ZO-1 antibody (red). Scale is approximately 5 µm.

D. Graph showing quantitation of the number of cilia/unit area along the ventricular surface of wild type (WT) and mutant *CWH43*$^{M533/A530}$ mice. Data shown are the mean ± SEM. * = *P*<0.0001, n=6, unpaired t-test.
