# Supplementary figures and images for: Deletions in CWH43 cause idiopathic normal pressure hydrocephalus
Source: EMBO Mol Med. 2021 Jan 18;13(3):e13249. doi: 10.15252/emmm.202013249 (PMC7933959; doi:10.15252/emmm.202013249)

Figure 1D

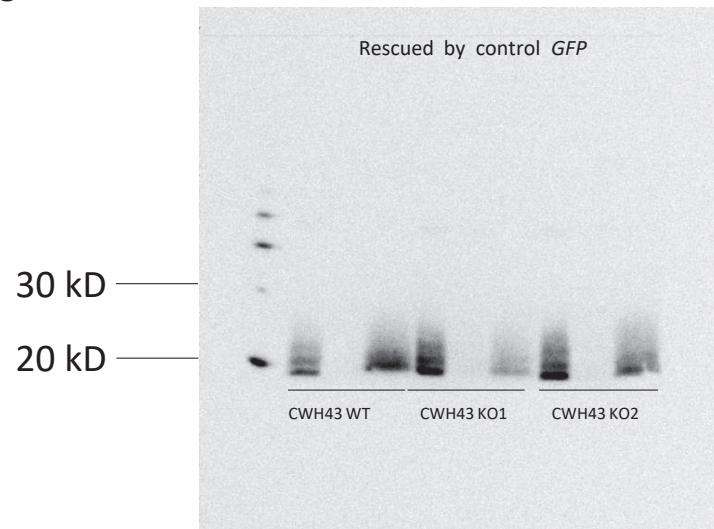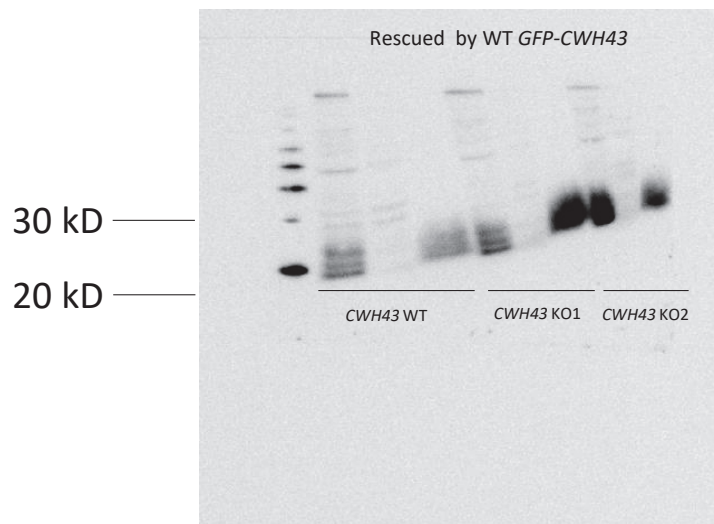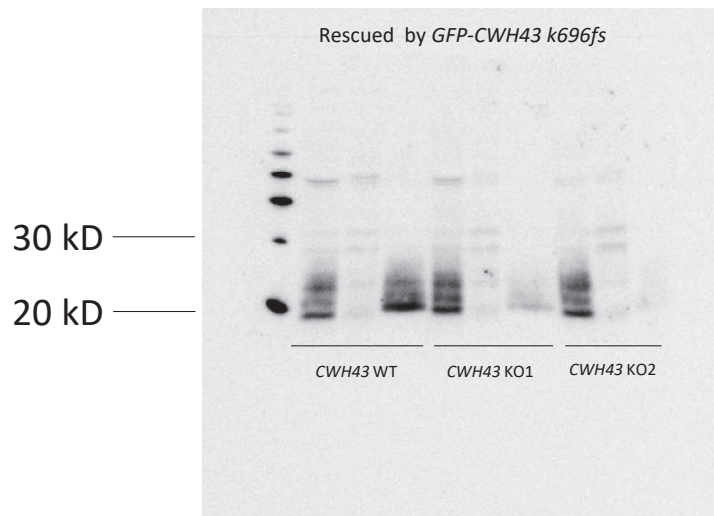

Supplement: Supplementary file 3 — Source Data for Figure 1 [file EMMM-13-e13249-s002.pdf]
