# Supplementary material for: Deletions in CWH43 cause idiopathic normal pressure hydrocephalus
Source: EMBO Mol Med. 2021 Jan 18;13(3):e13249. doi: 10.15252/emmm.202013249 (PMC7933959; doi:10.15252/emmm.202013249)
Supplement: Supplementary file 5 — Source Data for Figure 4 [file EMMM-13-e13249-s004.pdf]

Figure 4A

Cilated cell number /1500μm^2

	CWH43 $^{WT/WT}$	*CWH43* $^{M533/M533}$
	19	13
	18	15
	17	11
	17	14
Average	17.75	13.25
STDEVA	0.957427108	1.707825128
T.Test		*CWH43* $^{WT/WT}$ vs *CWH43* $^{M533/M533}$
P Value		0.003704513

Figure 4B

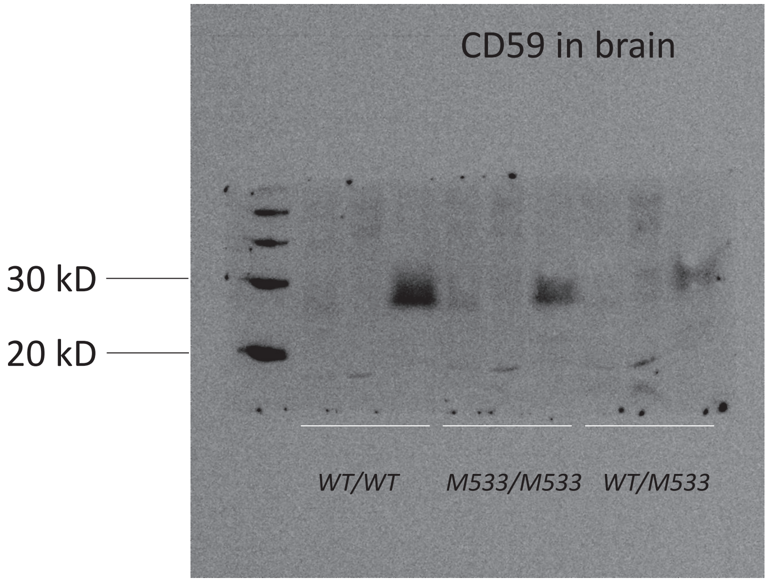
CD59 in brain
30 kD
20 kD
WT/WT
M533/M533
WT/M533

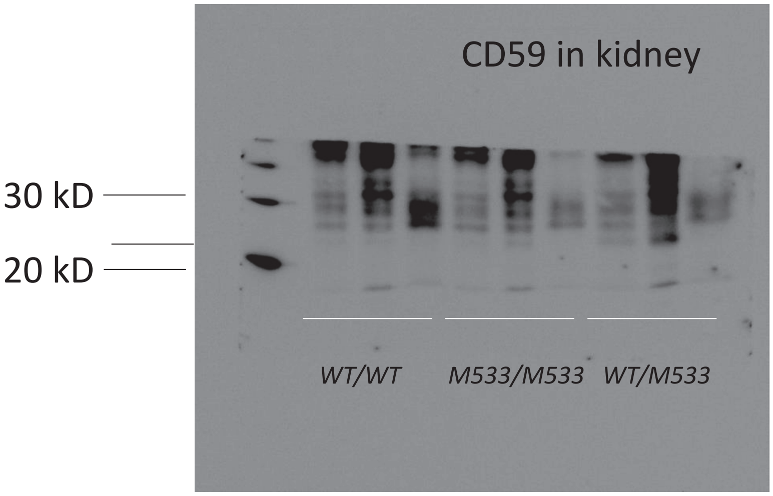
CD59 in kidney
30 kD
20 kD
WT/WT
M533/M533
WT/M533
